# Systems analysis of inflammatory bowel disease based on comprehensive gene information

**DOI:** 10.1186/1471-2350-13-25

**Published:** 2012-04-05

**Authors:** Satoru Suzuki, Takako Takai-Igarashi, Yutaka Fukuoka, Dennis P Wall, Hiroshi Tanaka, Peter J Tonellato

**Affiliations:** 1 Graduate School of Medical and Dental Sciences, Tokyo Medical and Dental University, 1-5-45 Yushima, Bunkyo-ku, Tokyo 113-8510, Japan; 2 Graduate School of Biomedical Science, Tokyo Medical and Dental University, 1-5-45 Yushima, Bunkyo-ku, Tokyo 113-8510, Japan; 3 Center for Biomedical Informatics, Harvard Medical School, 10 Shattuck Street, Boston, MA 02115, USA

**Keywords:** Inflammatory bowel disease (IBD), Disease related genes, Protein-protein interaction networks, GO based functional score, Interpretation of pathogenesis

## Abstract

**Background:**

The rise of systems biology and availability of highly curated gene and molecular information resources has promoted a comprehensive approach to study disease as the cumulative deleterious function of a collection of individual genes and networks of molecules acting in concert. These "human disease networks" (HDN) have revealed novel candidate genes and pharmaceutical targets for many diseases and identified fundamental HDN features conserved across diseases. A network-based analysis is particularly vital for a study on polygenic diseases where many interactions between molecules should be simultaneously examined and elucidated. We employ a new knowledge driven HDN gene and molecular database systems approach to analyze Inflammatory Bowel Disease (IBD), whose pathogenesis remains largely unknown.

**Methods and Results:**

Based on drug indications for IBD, we determined sibling diseases of mild and severe states of IBD. Approximately 1,000 genes associated with the sibling diseases were retrieved from four databases. After ranking the genes by the frequency of records in the databases, we obtained 250 and 253 genes highly associated with the mild and severe IBD states, respectively. We then calculated functional similarities of these genes with known drug targets and examined and presented their interactions as PPI networks.

**Conclusions:**

The results demonstrate that this knowledge-based systems approach, predicated on functionally similar genes important to sibling diseases is an effective method to identify important components of the IBD human disease network. Our approach elucidates a previously unknown biological distinction between mild and severe IBD states.

## Background

Inflammatory Bowel Disease (IBD) is a chronic disease of unknown etiology that causes inflammation and ulcer in intestinal mucosa. Although IBD is still much less prevalent in Japan than in Western countries, the number of Japanese IBD patients has rapidly increased in the last 20 years [1]. This rising trend, also observed in the Asia-Pacific region [2,3] indicates that IBD is rapidly becoming a world-wide disease. There are two major sub-categories of IBD: Crohn's disease (CD) and ulcerative colitis (UC) [4]. Although the pathogenesis of IBD is not fully explained, genetic factors are suggested to contribute to dysregulation of intestinal immunity, leading to gastrointestinal injury.

A genetic study of IBD was first reported in 1988 as an epidemiological study of CD patients [5]. Genome-wide scanning (GWS) studies have revealed nine IBD susceptibility loci (*IBD1-9*) [6] and one susceptibility gene (*NOD2*) [7]. Genome-Wide Association Studies (GWAS) and corresponding meta-analyses identified 71 susceptibility loci for CD [8] and 47 loci for UC [9]. Recently the genetic susceptibility to IBD was comprehensively reviewed in [10]. Another study based on and analysis of molecular pathways suggested a significant overlap between IBD and autoimmune disorder, type 1 diabetes, ankylosing spondylitis (AS), multiple sclerosis, asthma [11], and rheumatoid arthritis (RA) [12].

IBD medical treatment policy and drug selection is determined according to IBD 'severity' (mild, moderate, or severe) [4,13-15], which reflects the frequency of rectal bleedings and stools as well as mucosal appearances on sigmoidoscopy. Aminosalicylate (*e.g.*, Mesalazine) is a primary drug for the mild state, and anti-TNF antibody (*e.g.*, Infliximab) is a primary drug for the severe state. Both drugs act on inhibition of inflammatory cascades; however, while the anti-TNF antibody specifically interacts with TNF, aminosalicylate is similar to other members of nonsteroidal anti-inflammatory drugs (NSAIDs) having several molecular targets.

Recently, human diseases have been studied with a systems approach to address the need to understand the network of genes and molecules acting in concert to produce the pathogenesis and progression of the disease. A human disease network (HDN) is a representation of the etiology of the disease by protein-protein interaction (PPI) data and information. Goh *et al. *constructed a comprehensive HDN to cover all diseases and showed that essential human genes encoded hub proteins in the network [16]. Hase *et al. *investigated physical properties of the comprehensive HDN, and elucidated that there were extensive interconnections among middle-degree nodes that formed the backbone of the network [17]. Hase *et al. *also investigated drug-target genes in the HDN, and found significant preference of drug targets for middle- and low-degree nodes [17].

Wall *et al. *studied the HDN for autism. They constructed the network by genes related to autism and other neurological disorders in order to elucidate common features in the diseases [18]. They identified 154 genes not previously linked to autism. Based on this study, Wall *et al. *developed a web tool for comprehensive genetic annotation of diseases called "Genotator" [19]. Genotator provides an up-to-date and comprehensive collection of disease genes and a reliable gene-to-disease ranking for any disease. They showed that the integration of all the available databases gave a more complete picture than any one database alone, using Alzheimer disease as a case study.

The idea of Genotator inspired us to investigate IBD by a knowledge driven HDN gene and molecular database systems approach. Like autism and Alzheimer disease, IBD is known to be a complex disease with a large number of genes and molecules implicated in the etiology of the disease but without direct experimental evidence. A primary difference between our approach and Wall's is our use of available drugs, their targeted pathways and genes and related drug treatment information to highlight the more important genes to the disease (in this case IBD). We assume that current effective drug treatments and their targets provide essential information that will help identify key pathways important in the pathogenesis and progression of the disease. We collected genes related to IBD and its drugs, constructed a disease network with the genes, and investigated the functional similarity of drug targets to the putative IBD genes. The human disease network results demonstrate a new approach to characterizing IBD and its progression from early stage into the chronic and more malignant state.

## Methods

### Drug information on IBD and sibling diseases

We use the World Gastroenterology Organization [4] guidelines and IBD societies of USA [20], Europe [21-24], and Asia [2,3] to identify drugs for IBD and sibling diseases. Additional drug information was collected from PubMed and the Cochrane Library databases. The drug indications were investigated by databases: Package Inserts Database (in Japanese) [25] for Japanese drugs, FDA Approved Drug Products [26] for US drugs, and the electronic Medicines Compendium (eMC) [27] for UK drugs. Thereafter, target molecules and pathways of a drug were retrieved from the DrugBank database [28]. We then selected a target molecule of all the drugs (compendium of drugs are found in Table 1).

**Table 1:** A list of drugs for Inflammatory Bowel Disease and their indications

IBD state	Category	Drug name	Indication		
			
			US	UK	Japan
Mild	Amino-salicylate	Mesalazine	UC, CD	UC, CD	UC, CD
		
		Salazo-sulfapyridine	UC, CD, RA	UC, CD, RA	UC, CD, RA
		
		Olsalazine	UC	UC	-
		
		Balsalazide	UC	UC	-

Moderate	Immuno-modulators	Azathioprine	RA, GVHD	RA, SLE, DM, AICAH, PM, PV, PN, AHA ITP	GVHD, CD
		
		Tacrolimus	PTR	PTR	PTR, GVHD, MG, RA, LN, UC
	
	Corticosteroids	Budesonide (Entocort only)	CD	CD	-
		
		Prednisolone	RA, UC (chronic), Nephrotic Syndrome, Collagen Diseases, Fulminating SLE, Allergic Conditions, Bronchial Asthma, Acute Skin Diseases, Thrombocytopenia, Organ transplantation.		

Severe	Anti-TNF antibody	Infliximab	UC, CD, RA, PS, AS	UC, CD, RA, PS, AS	UC, CD, RA, PS, AS, BD
		
		Adalimumab	CD, RA, PS, AS	CD, RA, PS, AS	-
		
		Certolizumab	CD, RA	RA	-

Abbreviations: *CD *Crohn's disease, *UC *Ulcerative colitis, *RA* Rheumatoid artitis, *PS *Psoriasis (including *PS *Psoriatic arthritis), *AS *Ankylosing spondylitis, *BD *Bachet disease, *GVHD *Graft versus host disease, *SLE *Systemic lupus erythematosus, *DM *Dermatomyosis, *PM *Polymyositis, *AICAH *Auto-immune chronic active hepatitis, *PV *Pemphigus vulgaris, *PN *Polyarteritis nodosa, *AHA *Auto-immune haemolytic anaemia, *ITP *Idiopathic thrombocytopenic purpura, *MG *Myasthenia gravis, *LN *Lupus nephritis, *PTR *Platelet transfusion refractoriness

### Collection of disease genes from public databases

The basic gene association approach originated from Genotator [19] which collected evidence for the association of gene to disease from multiple databases and then scored the evidence thus providing an evidenced-based ranked list of genes associated with a given disease. Here, we collected IBD and sibling disease genes from HuGE Navigator, PharmGKB, GeneCards and Genetic Association Database (GAD). We downloaded the PharmGKB and GAD datasets and 'scraped' genes from the HuGE Navigator and GeneCards web applications. All data was selected in August, 2010. On the gene collection, we employed all the variation of synonymous names of IBD and sibling diseases. The collection of genes were then merged by matching EntrezGene IDs. If a database did not provide a gene's EntrezGene IDs, we used the DAVID bioinformatics tools [29,30] to retrieve the EntrezGene IDs.

### Protein-Protein Interaction network

A Protein-Protein Interaction (PPI) network was produced by STRING8 (October, 2010) using at least one experimental association result, information from PPI databases, including additional edges of one path (depth = 2), and with high confidence (confidence > 0.9). We used CYTOSCAPE [31] to visualize the PPI networks.

### Semantic similarity between two genes

We employed the same scoring as [32]. First, define

(1) IC(c)=-log(p(c))

where *p(c) *is the frequency of annotation of the term *c *and its children in the GO graph. Here, *Information Content (IC) *is a measure of how informative a term ***c ***is relative to other terms [33]. Second, define the similarity between two GO terms *c_1 _*and *c_2 _*as:

(2) $$SIM_{term}\left(c_{1},c_{2}\right)=\text{arg}\underset{c\in A\left(c_{1},c_{2}\right)}{\text{max}}\left(IC\left(c\right)\right)$$

where *A(c_1_,c_2_) *is the set of common ancestors of *c_1 _*and *c_2_. SIM_term_(c_1_,c_2_) *is defined as the most informative common ancestor of *c_1 _*and *c_2 _*[34,35]. Then, define the similarity between two GO term sets *A *and *B *(*SIM_termset_(A,B*)), assuming that the gene *g_A _*and the gene *g_B _*are annotated respectively with *n *and *m *GO terms as *A = {GO_1A_, GO_2A_,...,GO_nA_}*, *B = {GO_1B_, GO_2B_,...,GO_mB_}. SIM_termset_(A,B*) is defined as summation of the maximum similarities between a term in set *A *and any of terms in set *B*, normalized by summation of *IC *of the terms in set *A*:

(3) $$SIM_{termset}\left(A,B\right)=\frac{\overset{n}{\underset{i=1}{\sum }}\text{arg}\underset{GO\in B}{\text{max}}SIM_{term}\left(GO_{iA},GO\right)}{\overset{n}{\underset{i=1}{\sum }}\text{arg}\underset{GO\in B}{\text{max}}SIM_{term}\left(GO_{iA},GO\right)+\overset{n}{\underset{i=1}{\sum }}IC\left(GO_{iA}\right)}$$

Please note that *SIM_termset_(A, B) *is not equal to *SIM_termset_(B, A)*. Then the expected similarity *SSM *between the *g_A _*and the *g_B _*is defined as:

(4) $$SSM\left(g_{A},g_{B}\right)=\frac{SIM_{termset}\left(A,B\right)+SIM_{termset}\left(B,A\right)}{2}$$

(symbol for member of) 0[1]. A pseudo-code for *SSM *calculation is available in Additional file 1. We calculated the random distribution of the *SSM *score; 1,000 randomly collected human genes were tested against the drug targets for *IBDmild *and *IBDsevere *separately. Both distributions showed upper quartile threshold scores to be 0.5 (0.50760 and 0.50940 for *IBDmild *and *IBDsevere *respectively), and we determined that the upper quartile was best suited to define both those genes most likely closely associated with the disease but least likely to be unimportant. Consequently, we used the 0.5 threshold as criteria for our *SSM *scoring.

### Gene score for record frequency

Define *Record Frequency RF_g _*as the total frequency of records of a certain gene related to the sibling disease (*sib*) contained in the database (*db*):

(5) $$RF_{g}=\text{log}\left(\frac{\underset{j\in db}{\sum }\underset{i\in sib}{\sum }RF_{g}\left(i,j\right)}{N_{db}N_{sib}}\times \frac{N_{ref}}{DF_{g}}\right)$$

where *N_db _*is the total number of tested databases (four in this study: HugeNavigator, PharmGKB, GeneCards, and GAD), *N_sib _*is the total number of the sibling diseases, *N_ref _*is the total number of papers reporting on any human gene, and *DF_g _*is the frequency of papers reporting a certain gene. We calculated *DF_g _*and *N_ref _*by Entrez Programming Utilities provided by NCBI. When calculating *DF_g_*, we replaced a name of a certain gene with its synonyms obtained from a complete gene information table ("Homo_sapiens.gene_info") provided by NCBI. *N_ref _*was evaluated by the number of papers annotated with MeSH terms of "homo sapiens" and "gene" or "protein". *N_ref _*~2.3 million (Aug 10th, 2010).

### Gene expression data and analysis

We retrieved 12 data sets from GEO and 13 data sets from ArrayExpress by a query with keywords of "Crohn's disease, CD, ulcerative colitis, UC, inflammatory bowel disease, IBD, homo sapiens". After investigation of individual data sets, only three of them (GSE6731 [36], GSE9452 [37], and E-TABM-118 [38]) consist of patients with CD and UC and include patient information on medicinal drugs the patients took. Consistent with our fundamental approach, we classified the patients prescribed aminosalicylate into "patients in mild state" (*IBDmild) *and the patients prescribed anti-TNF antibody into "patients in severe state" (*IBDsevere)*. Since E-TABM-118 includes very few patients in the severe state, we focused on differential expressed gene analysis with GSE6731 and GSE9452 using the "samr" package in R [39] between *IBDmild *and *IBDsevere *patients and data sets. Genes of FDR < 0.05 were selected (2,549 genes from GSE6731 and 17 genes from GSE9452) and mapped the genes to our PPI network produced by STRING8 resulting in 28 genes from GSE6731 (listed in Additional file 2: Table S4) and no genes from GSE 9452 mapped to the PPI network.

## Results

### Determination of sibling diseases for the mild and severe states of IBD

As in autism spectrum disorder [18], we assumed genes common among the IBD network of sibling diseases would represent a molecular mechanism essential to all sibling diseases including IBD. The sibling diseases were determined as follows.

The World Gastroenterology Organization defines three categories of malignancy in IBD, mild, moderate, and severe states in its global guidelines for IBD treatment [4]. There is also clear categorization in drug indication to IBD according to the malignancy: aminosalicylate drugs for mild state patients, immunomodulator drugs and corticosteroid drugs for moderate state patients, and anti-TNF antibody drugs for severe state patients (Table 1). We then assumed the drugs provided the key to determine known sibling diseases for IBD. Table 1 shows diseases to which the same drugs as IBD are indicated in US, UK, and Japan. CD, UC, and RA are representative diseases treated with the drug of the mild state (aminosalicylate), while PS, AS, and BD are representative diseases treated using the drug of the severe state (anti-TNF antibody). Although the severe state also includes CD, UC, and RA, the three diseases were eliminated from the sibling group for the severe state (Table 1) to investigate the net difference between the mild and severe states. We therefore determined these diseases as the sibling diseases of IBD for its mild: *IBDmild *= {CD, UC, RA} and severe: *IBDsevere *= {PS, AS, BD} states (as used above). The moderate state was neglected from this study because we aimed at a comparison between the most extreme malignancy IBD states.

### Collection of disease genes from public databases

**Figure 1:**
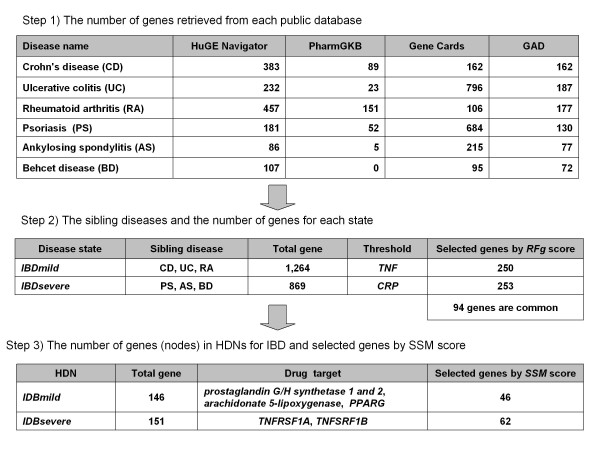
**The number of genes at each step of the analysis**.

Figure 1 shows the number of genes related to the two sibling diseases, *IBDmild *and *IBDsevere*. Genes for each sibling disease (1,264 for *IBDmild *and 869 for *IBDsevere*, Figure 1) were collected from the public databases: HugeNavigator, GeneCards, PharmGKB, and GAD. For each gene, *g*, we calculated the ranking score *RFg *by the frequency of the gene in databases and the frequency of the gene in PubMed according to equation (5). *RFg *was calculated for all genes associated with each sibling disease. We then determined a threshold score for each sibling disease used to exclude genes without significant evidence supporting their association with the sibling disease. To define the thresholds, we identified all genes in the two sibling disease gene sets which have verified association with IBD by direct literature review. We then scored all verified genes and chose the gene with lowest score in each sibling gene set (*TNF *for *IBDmild *and *CRP *for *IBDsevere*). Applying these thresholds to the *RFg *ranked list of sibling genes resulted in 250 and 253 genes for *IBDmild *and *IBDsevere *respectively. The intersection of the two sibling disease sets was 94 genes.

**Figure 2:**
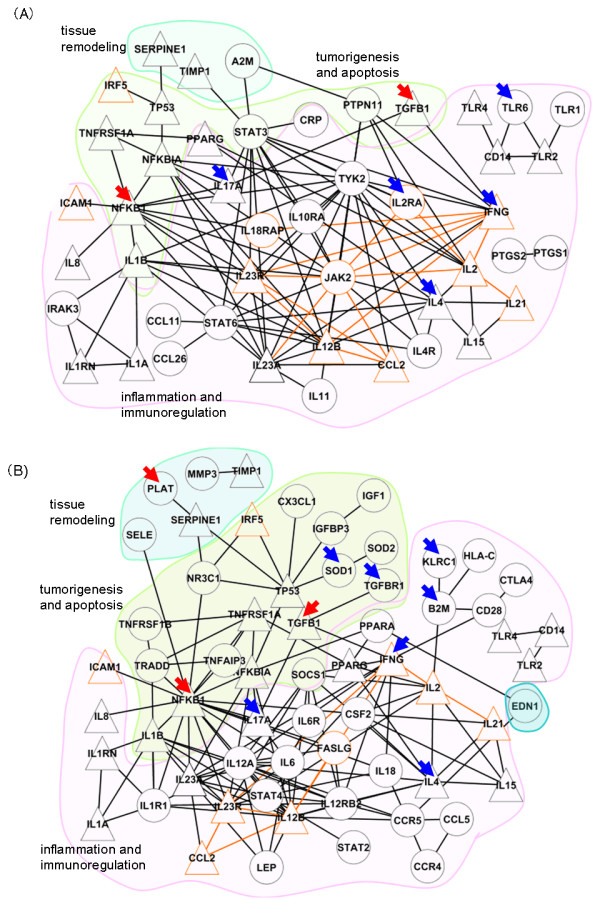
**Protein-protein interactions among the genes with high functional similarities to the drug targets**. (A) A network composed of functionally similar genes to *prostaglandin G/H synthetase 1 and 2*, *arachidonate 5-lipoxygenase*, and *PPARG*, all of which are drug targets for *IBDmild*. (B) A network composed of functionally similar genes to *TNF receptor 1A *and *1B*, which are drug targets for *IBDsevere*. In each network, a triangle node indicates a gene common to the HDNs of *IBDmild *and *IBDsevere*, a circle node indicates a gene specific either the HDN of *IBDmild *or *IBDsevere*. A node bordered with an orange line indicates a gene detected by GWAS. An edge connecting the GWAS genes is also highlighted in pale orange. A node marked with an arrow indicates a differentially expressed gene in GSE6731. A blue arrow indicates an up-regulated gene in *IBMmild*, while a red arrow indicates an up-regulated gene in *IBMsevere*. Both networks are divided into three portions by colored areas. The blue area indicates genes relate to tissue remodeling. The green area indicates genes relate to inflammation and immunoregulation. The rose area indicates genes relate to tumorigenesis and apoptosis.

All genes in the *IBDmild *and *IBDsevere *gene sets were tested for Protein-Protein Interactions (PPIs) with STRING8 [40] and those with at least one published experimental result and information from the PPI databases support were included in subsequent HDN analysis. A total of 146 (*IBDmild*) and 151 (*IBDsevere*) genes had at least one validated PPI relationship (Figure 1). We define the collective PPI networks as the "HDN" for IBD.

### Significant genes in *IBDmild *and *IBDsevere*

Our goal is to identify significant genes representing differences between *IBDmild *and *IBDsevere*. We focused on drugs that treat either *IBDmild *or *IBDsevere*. Aminosalylate was the drug specific to *IBDmild*, and anti-TNF antibody was the drug specific to *IBDsevere *(Table 1). In a cell, aminosalylate inhibits *PTGS1*, *PTGS 2*, *ALOX5*, and *PPARG*. These four genes were defined as drug targets for *IBDmild *in this study. On the other hand, anti-TNF antibody inhibits *TNF receptor 1A *and *1B*. These two genes were defined as drug targets for *IBDsevere*. We consider a gene to be functionally similar to these drug target genes as a significant gene potentially representing differences between *IBDmild *and *IBDsevere*. Such a functional similarity can be measured by a score provided by Gene Ontology (GO). There have been reports on application of the functional similarity score to the analysis of PPI networks [33-35]. We employed the GO-based similarity score (*SSM *in equation (4)) to our HDNs to highlight functional similarity of a gene to the drug targets.

Figure 2 shows the HDNs whose nodes were selected by the functional similarity of a gene to the drug targets. A node scored less than 0.5 (upper quartile of the random distribution of *SSM*) were eliminated from the HDNs. A triangular node indicates a gene belongs to both HDNs of *IBDmild *and *IBDsevere*, while a circular node represents a gene belongs to either HDN of *IBDmild *or *IBDsevere*. Additional file 3: Table S2 lists the genes consist of the HDNs. The similarity score in this study showed a mean score of 0.40050, with a standard deviation of 0.13942, a maximum score of 0.86640, a minimum score of 0.02972, and a upper quartile of 0.50760 against *IBDmild *drug targets, and a mean score of 0.37570, with a standard deviation of 0.17189, a maximum score of 0.91200, and a minimum score of 0.02417, and a upper quartile of 0.50940 against *IBDsevere *drug targets.

### Differences between the HDNs of *IBDmild *and *IBDsevere*

Table 2 shows the functional classification of genes in the HDNs of *IBDmild *and *IBDsevere*. Both HDNs consist of similar kinds of functional groups: i.e., inflammation, innate and acquired immune response, apoptosis, tumorigenesis, and tissue remodeling. However, the HDN of *IBDsevere *includes genes of tumorigenesis and apoptosis larger in number than the HDN of *IBDmild *(encircled by a green area in Figure 2(A) and 2(B)). The HDN of *IBDmild *also includes some genes of tumorigenesis and apoptosis, but the genes stay peripherally around a central gene group of inflammation and immunoregulation (encircled by a rose area). In contrast, the HDN of *IBDsevere *includes close interconnection among genes from the tumorigenesis and apoptosis group. This feature was not observed in the HDN of *IBDmild*.

**Table 2:** Functional classification of genes consist of HDNs for IBD

HDN	Functional category	Gene
*IBDmild*	immunoregulation	*ICAM1, IL2, IL23A, IL23R, IL4, CRP, IL11*, *IL2RA, IL4R, IRAK3, TYK2*
	
	innate immunity	*CD14, TLR2, TLR4, TLR1, TLR6, IFNG, IL8*
	
	inflammation and immunoregulation	*CCL2, IL12B, IL17, IL1A, IL1RN, NFKB1*, *NFKBIA, CCL11, CCL26, IL10RA, IL18RAP*
	
	inflammation and apoptosis	*IL1B, IRF5, TNFSF1A, IL15, JAK2, STAT6*
	
	cell growth, apoptosis and tumorigenesis	*TP53, PTPN11, STAT3, TGFB1*
	
	atherosclerosis and tissue remodeling	*SERPINE1, TIMP1, A2M*
	
	diabetes related	*PPARG*
	
	prostaglandin biosynthesis	*PTGS1, PTGS2*

*IBDsevere*	immunoregulation	*ICAM1, IL2, IL23A, IL23R, IL4, B2M, CD28, CX3CL1, CTLA4, HLA-C, KLRC1, IL12A*, *IL12RB2, STAT2, STAT4*
	
	innate immunity	*CD14, TLR2, TLR4, IFNG, IL8*
	
	inflammation and immunoregulation	*CCL2, IL12B, IL17, IL1A, IL1RN, NFKB1*, *NFKBIA, CCL5, CSF2, IL1R1, IL6, IL6R, IL18, NR3C1*
	
	inflammation and apoptosis	*IL1B, IRF5, TNFSF1A, IL15*
	
	apoptosis	*FASLG, TNFAIP3, TNFRSF1B, TRADD*
	
	cell growth and tumorigenesis	*TP53, IGF1, IGFBP3, TGFB1, TGFBR1, SOCS1*
	
	aging and tumorigenesis	*SOD1, SOD2*
	
	atherosclerosis and tissue remodeling	*SERPINE1, TIMP1, EDN1, MMP3, PLAT, SELE*
	
	diabetes related	*PPARG, LEP, PPARA*

We annotated the genes by their functions according to EntrezGene database.

Both in the HDNs of *IBDmild *and *IBDsevere*, some genes of inflammation and immunoregulation have highly condensed interactions with surrounding genes. Interestingly, numbers of them were detected by the GWAS studies [8,9] (Additional file 4: Table S3). These genes (*IL2*, *IL12B*, *IL23R*, *IFNG*, and *JAK2*) indicate significant functionalities in the IBD pathogenesis. Besides, differentially expressed gene between *IBDmild *and *IBDsevere *are also included in the condensed interconnections of genes. They are *IFNG, IL4*, *IL17A*, *NFKB1*, and *TGFB1 *(indicated by either blue or red arrow in Figure 2), selected by SAM statistics (FDR < 0.05) on gene expression data of GSE6731. While the genes of inflammation and immunoregulation were up-regulated in *IBDmild *(*IFNG*, *IL4*, and *IL17A*, indicated by blue arrows), the genes of tumorigenesis and apoptosis were up-regulated in *IBDsevere *(*NFKB1 *and *TGFB1*, indicated by red arrows).

### The molecular picture of the progression of IBD

IBD is characterized by the progression from early stage into chronic and more malignant states. The characteristic is widely used in the clinical treatment of IBD, but its molecular processes remains unclear. This study provides a molecular picture for the progression of IBD. The molecular picture tells us a difference between the mild and severe states, two extreme states in the progression of IBD. The biological details are described in "Discussion".

## Discussion

Our knowledge driven HDN gene and molecular database systems approach consists of the following steps: 1) Determination of sibling diseases for IBD based on drug information, 2) Collection of IBD and sibling disease genes from multiple databases, 3) Scoring the disease genes by evidence-based ranking weighted by "the frequency in databases" and "the frequency in PubMed", 4) Evaluation of the disease genes for Protein-Protein Interaction relations, and 5) Investigation of GO-based functional similarity of drug targets to the putative IBD genes. We summarized the criteria for our selection of genes specific to *IBDmild *and *IBDsevere *in Table 3. Our results may lead to an elucidation for IBD pathogenesis that remains largely unknown.

**Table 3:** Criteria for selection of genes specific to *IBDmild *and *IBDsevere*

Criteria for selection	No. of *IBDmild *genes	No. of *IBDsevere *genes
(1) Collect IBD-associated genes from databases for human diseases.	1,264	869

(2) Rank the genes by the frequency of the IBD-association in the databases (equation (5)). Cut off the genes falling short of the lowest frequency of a gene validated its disease association by literatures (*TNF *for *IBDmild *and *CRP *for *IBDsevere*).	250	253

(3) Select the genes interconnected by PPI evidence.	146	151

(4) Rank the genes by GO based similarity score (*SSM*, equation (4)) to IBD drug targets. Cut off the genes under the upper quartile of the random distribution of *SSM *scores against IBD drug targets.	46	62

Sibling diseases, closely aligned to a complex disease such as IBD, provide a novel opportunity to use comprehensive omics data to identify a core biochemical or treatment pathway not previously identified in medical biology. If correctly defined, a collection of sibling diseases can 'cover' the entire pathophysiological process of a target disease more completely than any one disease. Wall *et al. *determined autism sibling diseases based on commonly involved genes [18]. We defined IBD sibling diseases based on drug indications. Drugs act on changing pathogenic states of a disease. Accordingly, it is highly likely that the sibling diseases share a common molecular mechanism. This approach may be generalizable if drugs acting on sibling disease states, are available across the developmental progression of a given central disease.

Figure 2 indicates that the HDNs for IBD change according to the state of the disease progression. Our results show that *JAK2 *and *STAT3*, which are known to be associated with IBD [41], are specific to the HDN of *IBDmild *(Figure 2(A)) [10]. These genes form the central part of the HDN of *IBDmild *and have links to pro-inflammatory genes (*IL1A/1B*, *IL12B*, *IL17A*, *and IL23A/R*), which are common in both HDNs. These suggest that *JAK2 *and *STAT3 *are key factors in the early stage of IBD pathology. On the other hand, the HDN of *IBDsevere *(Figure 2(B)) indicates a relation of IBD to more malignant diseases like cancer. Patients with long-standing IBD have an increased risk of developing colorectal cancer [42]. Such a transition in functional classes of genes was also observed with differential expressed genes between *IBDmild *and *IBDsevere *obtained by GSE6731 (nodes with arrows in Figure 2).

The HDNs of *IBDmild *and *IBDsevere *include 13 genes obtained by GWAS reported in [8,9]. The 13 genes are highlighted by colored borders in orange in Figure 2 and listed in boldfaced in Additional file 3: Table S2. Most of the genes are interconnected with each other except *ICAM1 *and *IRF5*. All interconnected genes belong to the early response of inflammation, which includes cytokines, chemokines, receptors, and cellular signaling molecules. The other two genes, *ICAM1 *and *IRF5*, belong to the late response of inflammation, i.e., enhancement of immune response. Our HDNs illustrate what molecules intervene between the two sibling disease pathways. Both in the HNDs of *IBDmild *and *IBDsevere*, *TP53 *and *NFKB1*/*NFKBIA *are common to both disease states, indicating that the transcriptional regulation intervenes between the early (*IBDmild*) and late (*IBDsevere*) responses. In this way, our HDN analysis and approach helps to clarify the molecular and therefore disease implications of GWAS candidate genes.

A recent comprehensive review of molecular pathways for IBD pathogenesis [10] supports characteristic genes in our HDNs of *IBDmild *and *IBDsevere*. The genes characteristic of the HDN of *IBDmild *(*Jak2*, *Stat3*, and *IL23*) belong to Th17-cell differentiation in [10], and the genes characteristic of the HDN of *IBDsevere *(*IL12*, *IFN-gamma*, *IL18 *and *FASLG*) belong to Th1-cell driven responses in [10,36]. The physiological balance between Th1 and Th17 may be deteriorated by environmental factors such as intestinal bacteria stress, which eventually leads to autoimmune responses composing IBD ("hygiene hypothesis" introduced by Strachan [43]). Our HDNs suggest a transition from Th17 to Th1 dominancy along with progression of malignancy [44]. Our approach stratifying the disease-related genes into *IBDmild *and *IBDsevere *enables us to infer a clinically significant transition of a state of a disease such as the Th1/Th17 transition.

## Conclusions

In this study, we employed a knowledge driven human disease systems approach to analyze IBD, whose pathogenesis remains largely unknown. Based on drug indications for IBD, we determined two sibling disease states of IBD (mild and severe). After ranking the genes by the frequency of the records, we obtained 250 and 253 genes for *IBDmild *and *IBDsevere*, respectively. We calculated functional similarities of these genes with IBD drug targets and drew their interactions as PPI networks we later defined as the two sibling disease HDNs. The HDNs revealed biological and clinical insights into the molecular differences between *IBDmild *and *IBDsevere*. The results demonstrated that knowledge annotation of sibling disease HDNs with focus on high similarity genes is an effective approach to identify common genes and pathways important to the complex disease network.

## Abbreviations

AS: Ankylosing spondylitis; BD: Bechet's disease; CD: Crohn's disease; IBD: Inflammatory bowel disease; PS: Psoriasis; RA: Rheumatoid arthritis; UC: Ulcerative colitis; GO: Gene ontology; GWS: Genome-wide scanning; GWAS: Genome-wide association studies; PPI: Protein-protein interaction.

## Competing interests

This research received no specific grant from any funding agency in the public, commercial, or not-for-profit sectors.

## Authors' contributions

SS conceived the study plan and performed the gene analysis with guidance from TTI and YF. PJT and DPW inspired an idea of aggregating gene information from public databases. TTI developed the programs for the gene analysis and data management. PJT and HT directed the study. SS, TTI and YF drafted the manuscript and all authors read and approved the final manuscript.

## Pre-publication history

The pre-publication history for this paper can be accessed here:

http://www.biomedcentral.com/1471-2350/13/25/prepub

## Supplementary Material

Additional file 1 **Pseudo-code for SSM calculation**. Click here for file

Additional file 2 **Table S4. Differential expressed genes between *IBDmild *and *IBDsevere***. Resulted 28 genes from GSE6731: differentially expressed between *IBDmild *and *IBDsevere *(FDR < 0.05) and mapped to our PPI network produced by STRING8. Click here for file

Additional file 3 **Table S2. Genes consisting of the HDNs**. Genes consisting of the HDNs of Figure 2 are listed in this Table. These genes were selected when being functionally similar to the drug targets of each state (*SSM *> 0.5), as well as when having PPI connections among themselves. Accordingly, the genes "common to both HDNs" are different between *IBDmild *and *IBDsevere*, because *SSM *scoring is different between the HDNs. Genes are boldfaced when they are susceptibility loci for IBD detected by GWAS [8,9]. Genes are underlined when they are selected as differential expressed genes by SAM statistics (FDR < 0.05) [39] with GSE6731. Abbreviations for gene names are lilsted in Additional file 5: Table S1. Click here for file

Additional file 5 **Table S1. Abbreviations for gene names**. All the abbreviations for gene names used in this manuscript are listed. Click here for file

Additional file 4 **Table S3. Bond order of susceptibility gene for IBD detected by GWAS**. GWAS susceptibility genes [8,9] are listed with their bond order in HDNs of *IBDmild *and *IBDsevere*. Click here for file

## References

[B1] Asakura K Nishiwaki Y Inoue N Hibi T Watanabe M Takebayashi T Prevalence of ulcerative colitis and Crohn's disease in Japan J Gastroenterol 2009 44 7 659 665 10.1007/s00535-009-0057-3 19424654

[B2] Ouyang Q Tandon R Goh KL Pan GZ Fock KM Fiocchi C Lam SK Xiao SD Management consensus of inflammatory bowel disease for the Asia-Pacific region J Gastroenterol Hepatol 2006 21 12 1772 1782 10.1111/j.1440-1746.2006.04674.x 17074013

[B3] Ooi CJ Fock KM Makharia GK Goh KL Ling KL Hilmi I Lim WC Kelvin T Gibson PR Gearry RB The Asia-Pacific consensus on ulcerative colitis J Gastroenterol Hepatol 2010 25 3 453 468 10.1111/j.1440-1746.2010.06241.x 20370724

[B4] Bernstein CN Fried M Krabshuis JH Cohen H Eliakim R Fedail S Gearry R Goh KL Hamid S Khan AG World Gastroenterology Organization Practice Guidelines for the diagnosis and management of IBD in 2010 Inflamm Bowel Dis 2010 16 1 112 124 19653289 10.1002/ibd.21048

[B5] Tysk C Lindberg E Jarnerot G Floderus-Myrhed B Ulcerative colitis and Crohn's disease in an unselected population of monozygotic and dizygotic twins. A study of heritability and the influence of smoking Gut 1988 29 7 990 996 10.1136/gut.29.7.990 3396969 PMC1433769

[B6] Rodriguez-Bores L Fonseca GC Villeda MA Yamamoto-Furusho JK Novel genetic markers in inflammatory bowel disease World J Gastroenterol 2007 13 42 5560 5570 17948929 10.3748/wjg.v13.i42.5560 PMC4172734

[B7] Hampe J Frenzel H Mirza MM Croucher PJ Cuthbert A Mascheretti S Huse K Platzer M Bridger S Meyer B Evidence for a NOD2-independent susceptibility locus for inflammatory bowel disease on chromosome 16p Proc Natl Acad Sci USA 2002 99 1 321 326 10.1073/pnas.261567999 11752413 PMC117559

[B8] Franke A McGovern DP Barrett JC Wang K Radford-Smith GL Ahmad T Lees CW Balschun T Lee J Roberts R Genome-wide meta-analysis increases to 71 the number of confirmed Crohn's disease susceptibility loci Nat Genet 2010 42 12 1118 1125 10.1038/ng.717 21102463 PMC3299551

[B9] Anderson CA Boucher G Lees CW Franke A D'Amato M Taylor KD Lee JC Goyette P Imielinski M Latiano A Meta-analysis identifies 29 additional ulcerative colitis risk loci, increasing the number of confirmed associations to 47 Nat Genet 2011 43 3 246 252 10.1038/ng.764 21297633 PMC3084597

[B10] Khor B Gardet A Xavier RJ Genetics and pathogenesis of inflammatory bowel disease Nature 2011 474 7351 307 317 10.1038/nature10209 21677747 PMC3204665

[B11] Torkamani A Topol EJ Schork NJ Pathway analysis of seven common diseases assessed by genome-wide association Genomics 2008 92 5 265 272 10.1016/j.ygeno.2008.07.011 18722519 PMC2602835

[B12] Heap GA van Heel DA The genetics of chronic inflammatory diseases Hum Mol Genet 2009 18 (R1):R101 R106 19297396 10.1093/hmg/ddp001

[B13] Truelove SC Witts LJ Cortisone in ulcerative colitis; final report on a therapeutic trial Br Med J 1955 2 4947 1041 1048 10.1136/bmj.2.4947.1041 13260656 PMC1981500

[B14] Hanauer SB Sandborn W Management of Crohn's disease in adults Am J Gastroenterol 2001 96 3 635 643 10.1111/j.1572-0241.2001.03671.x 11280528

[B15] Kornbluth A Sachar DB Ulcerative colitis practice guidelines in adults: American College Of Gastroenterology, Practice Parameters Committee Am J Gastroenterol 2010 105 3 501 523 quiz 524 10.1038/ajg.2009.727 20068560

[B16] Goh KI Cusick ME Valle D Childs B Vidal M Barabasi AL The human disease network Proc Natl Acad Sci USA 2007 104 21 8685 8690 10.1073/pnas.0701361104 17502601 PMC1885563

[B17] Hase T Tanaka H Suzuki Y Nakagawa S Kitano H Structure of protein interaction networks and their implications on drug design PLoS Comput Biol 2009 5 10 e1000550 10.1371/journal.pcbi.1000550 19876376 PMC2760708

[B18] Wall DP Esteban FJ Deluca TF Huyck M Monaghan T Velez de Mendizabal N Goni J Kohane IS Comparative analysis of neurological disorders focuses genome-wide search for autism genes Genomics 2009 93 2 120 129 10.1016/j.ygeno.2008.09.015 18950700

[B19] Wall DP Pivovarov R Tong M Jung JY Fusaro VA DeLuca TF Tonellato PJ Genotator: a disease-agnostic tool for genetic annotation of disease BMC Med Genomics 2010 3 50 10.1186/1755-8794-3-50 21034472 PMC2990725

[B20] Lichtenstein GR Hanauer SB Sandborn WJ Management of Crohn's disease in adults Am J Gastroenterol 2009 104 2 465 483 quiz 464, 484 10.1038/ajg.2008.168 19174807

[B21] Dignass A Van Assche G Lindsay JO Lemann M Soderholm J Colombel JF Danese S D'Hoore A Gassull M Gomollon F The second European evidence-based Consensus on the diagnosis and management of Crohn's disease: Current management J Crohns Colitis 2010 4 1 28 62 21122489 10.1016/j.crohns.2009.12.002

[B22] Van Assche G Dignass A Panes J Beaugerie L Karagiannis J Allez M Ochsenkuhn T Orchard T Rogler G Louis E The second European evidence-based Consensus on the diagnosis and management of Crohn's disease: Definitions and diagnosis J Crohns Colitis 2010 4 1 7 27 21122488 10.1016/j.crohns.2009.12.003

[B23] Stange EF Travis SP Vermeire S Reinisch W Geboes K Barakauskiene A Feakins R Flejou JF Herfarth H Hommes DW European evidence-based Consensus on the diagnosis and management of ulcerative colitis: Definitions and diagnosis J Crohns Colitis 2008 2 1 1 23 21172194 10.1016/j.crohns.2007.11.001

[B24] Travis SP Stange EF Lemann M Oresland T Bemelman WA Chowers Y Colombel JF D'Haens G Ghosh S Marteau P European evidence-based Consensus on the management of ulcerative colitis: Current management J Crohns Colitis 2008 2 1 24 62 21172195 10.1016/j.crohns.2007.11.002

[B25] Package Inserts Database http://www.pmda.go.jp/english/service/package.html

[B26] Drugs@FDA http://www.accessdata.fda.gov/scripts/cder/drugsatfda

[B27] Electronic Medicines Compendium (eMC) http://www.medicines.org.uk/EMC/default.aspx

[B28] Knox C Law V Jewison T Liu P Ly S Frolkis A Pon A Banco K Mak C Neveu V DrugBank 3.0: a comprehensive resource for 'omics' research on drugs Nucleic Acids Res 2011 39 Database D1035 D1041 21059682 10.1093/nar/gkq1126 PMC3013709

[B29] da Huang W Sherman BT Lempicki RA Systematic and integrative analysis of large gene lists using DAVID bioinformatics resources Nat Protoc 2009 4 1 44 57 19131956 10.1038/nprot.2008.211

[B30] Dennis G Jr Sherman BT Hosack DA Yang J Gao W Lane HC Lempicki RA DAVID: Database for Annotation, Visualization, and Integrated Discovery Genome Biol 2003 4 5 P3 10.1186/gb-2003-4-5-p3 12734009 PMC193660

[B31] Shannon P Markiel A Ozier O Baliga NS Wang JT Ramage D Amin N Schwikowski B Ideker T Cytoscape: a software environment for integrated models of biomolecular interaction networks Genome Res 2003 13 11 2498 2504 10.1101/gr.1239303 14597658 PMC403769

[B32] Accetturo M Creanza TM Santoro C Tria G Giordano A Battagliero S Vaccina A Scioscia G Leo P Finding new genes for non-syndromic hearing loss through an in silico prioritization study PLoS One 2010 5 9 e12742 10.1371/journal.pone.0012742 20927407 PMC2946934

[B33] Wang JZ Du Z Payattakool R Yu PS Chen CF A new method to measure the semantic similarity of GO terms Bioinformatics 2007 23 10 1274 1281 10.1093/bioinformatics/btm087 17344234

[B34] Louie B Higdon R Kolker E A statistical model of protein sequence similarity and function similarity reveals overly-specific function predictions PLoS One 2009 4 10 e7546 10.1371/journal.pone.0007546 19844580 PMC2760442

[B35] Yu H Jansen R Stolovitzky G Gerstein M Total ancestry measure: quantifying the similarity in tree-like classification, with genomic applications Bioinformatics 2007 23 16 2163 2173 10.1093/bioinformatics/btm291 17540677

[B36] Wu F Dassopoulos T Cope L Maitra A Brant SR Harris ML Bayless TM Parmigiani G Chakravarti S Genome-wide gene expression differences in Crohn's disease and ulcerative colitis from endoscopic pinch biopsies: insights into distinctive pathogenesis Inflamm Bowel Dis 2007 13 7 807 821 10.1002/ibd.20110 17262812

[B37] Olsen J Gerds TA Seidelin JB Csillag C Bjerrum JT Troelsen JT Nielsen OH Diagnosis of ulcerative colitis before onset of inflammation by multivariate modeling of genome-wide gene expression data Inflamm Bowel Dis 2009 15 7 1032 1038 10.1002/ibd.20879 19177426

[B38] Csillag C Nielsen OH Borup R Nielsen FC Olsen J Clinical phenotype and gene expression profile in Crohn's disease Am J Physiol Gastrointest Liver Physiol 2007 292 1 G298 G304 16959948 10.1152/ajpgi.00321.2006

[B39] Tusher VG Tibshirani R Chu G Significance analysis of microarrays applied to the ionizing radiation response Proc Natl Acad Sci USA 2001 98 9 5116 5121 10.1073/pnas.091062498 11309499 PMC33173

[B40] Jensen LJ Kuhn M Stark M Chaffron S Creevey C Muller J Doerks T Julien P Roth A Simonovic M STRING 8--a global view on proteins and their functional interactions in 630 organisms Nucleic Acids Res 2009 37 Database D412 D416 18940858 10.1093/nar/gkn760 PMC2686466

[B41] Lees CW Satsangi J Genetics of inflammatory bowel disease: implications for disease pathogenesis and natural history Expert Rev Gastroenterol Hepatol 2009 3 5 513 534 10.1586/egh.09.45 19817673

[B42] Xie J Itzkowitz SH Cancer in inflammatory bowel disease World J Gastroenterol 2008 14 3 378 389 10.3748/wjg.14.378 18200660 PMC2679126

[B43] Strachan DP Family size, infection and atopy: the first decade of the "hygiene hypothesis" Thorax 2000 55 Suppl 1 S2 S10 10943631 10.1136/thorax.55.suppl_1.s2 PMC1765943

[B44] Rizzo A Pallone F Monteleone G Fantini MC Intestinal inflammation and colorectal cancer: A double-edged sword? World J Gastroenterol 2011 17 26 3092 3100 21912451 10.3748/wjg.v17.i26.3092 PMC3158408

