# Supplementary material for: Systems analysis of inflammatory bowel disease based on comprehensive gene information
Source: BMC Med Genet. 2012 Apr 5;13:25. doi: 10.1186/1471-2350-13-25 (PMC3368714; doi:10.1186/1471-2350-13-25)
Supplement: Additional file 1 — Pseudo-code for SSM calculation. [file 1471-2350-13-25-S1.DOC]

**Supplemental Data**

Pseudo-code for *SSM* calculation.

* eq.(1), eq.(2), eq.(3) , and eq.(4). indicate equations in the section “**Methods**”.

**procedure SIM**;

**INPUT**:

ic[c_i_]: the array of IC score of the GO Term c_i_ calculated using eq.(1),

sim_term[c_i_,c_j_]: the array of SIM_term_ score between two GO terms c_i_ and c_j_ calculated using eq.(2),

goa[g_i_]: the array of the list of GO terms (c_1_,c_2_,...,c_j_) annotated to the gene g_i_. This array is made out of the GOA file obtained from GO homepage,

ppi_gene[i]: the array of the genes constructing the PPI networks,

drug_gene[i]: the array of the drug-target genes;

**OUTPUT**:

sim_termset[g_i_,g_j_]: the array of SIM_termset_ score between two sets of GO terms annotated to the gene g_i_ and to the gene g_j_ calculated using eq.(3);

**FOR** g_i_ in index of ppi_gene **DO**

**FOR** g_j_ in index of drug_gene **DO**

**FOR** c_n_ in GO term list of goa[g_i_] **DO**

ic_sum_gi_ := ic_sum_gi_ +ic[c_n_];

**FOR** c_m_ in GO term list of goa[g_j_] **DO**

**IF** m=1

**THEN** sim_max_cn_ := sim_term(c_n_,c_m_);

**ELSE IF** sim_max_cn_ < sim_term(c_n_,c_m_)

**THEN** sim_max_cn_ := sim_term(c_n_,c_m_);

**ENDIF**

**ENDFOR**

sim_max_sum_gigj_ := sim_max_sum_gigj_ + sim_max_cn_;

**ENDFOR**

sim_termset(g_i_,g_j_):= sim_max_sum_gigj_ / (sim_max_sum_gigj_ + ic_sum_gi_);

**ENDFOR**

**ENDFOR**

**procedure SSM;**

**INPUT**:

sim_termset[g_i_,g_j_]: the array of SIM_termset_ score between two sets of GO terms annotated to the gene g_i_ and to the gene g_j_ calculated using eq.(3);

**OUTPUT**:

ssm[g_i_,g_j_]: the array of SSM score between two genes g_i_ and g_j_ calculated using eq.(4);

**FOR** g_i_ and g_j_ in index of sim_termset **DO**

ssm[g_i_, g_j_] := (sim_termset[g_i_,g_j_] + sim_termset[g_j_,g_i_]) / 2;

**ENDFOR**
