# Supplementary material for: Systems analysis of inflammatory bowel disease based on comprehensive gene information
Source: BMC Med Genet. 2012 Apr 5;13:25. doi: 10.1186/1471-2350-13-25 (PMC3368714; doi:10.1186/1471-2350-13-25)
Supplement: Additional file 2 — Table S4. Differential expressed genes between IBDmild and IBDsevere. Resulted 28 genes from GSE6731: differentially expressed between IBDmild and IBDsevere (FDR < 0.05) and mapped to our PPI network produced by STRING8. [file 1471-2350-13-25-S2.DOC]

**Supplemental Table S4**

**Differential expressed genes between *IBDmild* and *IBDsevere*.**

Resulted 28 genes from GSE6731: differentially expressed between *IBDmild* and *IBDsevere* (FDR<0.05) and mapped to our PPI network produced by STRING8.

| up-regulated in *IBDmild* | up-regulated in *IBDsevere* |
| --- | --- |
| *ADAM17* | *APOE* |
| *ALB* | *DARC* |
| *B2M* | *NFKB1* |
| *DEFB1* | *PLAT* |
| *FAS* | *SST* |
| *FCGR2A* | *TGFB1* |
| *GGH* |  |
| *IFNA2* |  |
| *IFNG* |  |
| *IL17A* |  |
| *IL1R2* |  |
| *IL2RA* |  |
| *IL4* |  |
| *KIAA1109* |  |
| *KIR2DL1* |  |
| *KLRC1* |  |
| *MTRR* |  |
| *RUNX1* |  |
| *SOD1* |  |
| *TGFBR1* |  |
| *TLR6* |  |
| *ZNF148* |  |
