# Supplementary material for: Systems analysis of inflammatory bowel disease based on comprehensive gene information
Source: BMC Med Genet. 2012 Apr 5;13:25. doi: 10.1186/1471-2350-13-25 (PMC3368714; doi:10.1186/1471-2350-13-25)
Supplement: Additional file 3 — Table S2. Genes consisting of the HDNs. Genes consisting of the HDNs of Figure 2 are listed in this Table. These genes were selected when being functionally similar to the drug targets of each state (SSM > 0.5), as well as when having PPI connections among themselves. Accordingly, the genes "common to both HDNs" are different between IBDmild and IBDsevere, because SSM scoring is different between the HDNs. Genes are boldfaced when they are susceptibility loci for IBD detected by GWAS [8,9]. Genes are underlined when they are selected as differential expressed genes by SAM statistics (FDR < 0.05) [39] with GSE6731. Abbreviations for gene names are lilsted in Additional file 5: Table S1. [file 1471-2350-13-25-S3.DOC]

**Supplemental Table S2**

| PPI network | Gene |
| --- | --- |
| specific to HDN of *IBDmild* | *A2M, CCL11, CCL26, CRP, IRAK3,* ***JAK2****,* ***IL2RA****, IL4R, IL10RA, IL11,* ***IL18RAP****, PTGS1, PTGS2, PTPN11,* ***STAT3****, STAT6, TLR1,* *TLR6, TYK2* |
| specific to HDN of *IBDsevere* | *B2M, CCL5, CCR4, CCR5, CD28, CSF2, CTLA4, CX3CL1, EDN1,* ***FASLG****,　HLA-C, IGF1, IGFBP3, IL1R1, IL6, IL6R, IL12A, IL12RB2, IL18, KLRC1, LEP, MMP3, NR3C1, PLAT, PPARA, SELE, SOCS1, SOD1, SOD2, STAT2, STAT4, TGFBR1, TNFAIP3, TNFRSF1B, TRADD* |
| common to both HDNs of *IBDmild* and *IBDsevere* | ***CCL2****, CD14,* ***ICAM1****,* ***IFNG,*** *IL1A, IL1B, IL1RN,* ***IL2****, IL4, IL8,* ***IL12B****, IL15, IL17A,* ***IL21****, IL23A,* ***IL23R****,* ***IRF5****, NFKB1, NFKBIA, PPARG, SERPINE1, TGFB1, TIMP1, TLR2, TLR4, TNFRSF1A, TP53* |

Genes consisting of the HDNs of Figure 2 are listed in this Table. These genes were selected when being functionally similar to the drug targets of each state (*SSM* > 0.5), as well as when having PPI connections among themselves. Accordingly, the genes “common to both HDNs” are different between *IBDmild* and *IBDsevere*, because *SSM* scoring is different between the HDNs. Genes are boldfaced when they are susceptibility loci for IBD detected by GWAS . Genes are underlined when they are selected as differential expressed genes by SAM statistics (FDR<0.05) [3] with GSE6731. Abbreviations for gene names are listed in Additional file 5: Table S1.

1. Franke A, McGovern DP, Barrett JC, Wang K, Radford-Smith GL, Ahmad T, Lees CW, Balschun T, Lee J, Roberts R *et al*: **Genome-wide meta-analysis increases to 71 the number of confirmed Crohn's disease susceptibility loci**. *Nat Genet* 2010, **42**(12):1118-1125.

2. Anderson CA, Boucher G, Lees CW, Franke A, D'Amato M, Taylor KD, Lee JC, Goyette P, Imielinski M, Latiano A *et al*: **Meta-analysis identifies 29 additional ulcerative colitis risk loci, increasing the number of confirmed associations to 47**. *Nat Genet* 2011, **43**(3):246-252.

3. Tusher VG, Tibshirani R, Chu G: **Significance analysis of microarrays applied to the ionizing radiation response**. *Proc Natl Acad Sci U S A* 2001, **98**(9):5116-5121.
