# Supplementary material for: Systems analysis of inflammatory bowel disease based on comprehensive gene information
Source: BMC Med Genet. 2012 Apr 5;13:25. doi: 10.1186/1471-2350-13-25 (PMC3368714; doi:10.1186/1471-2350-13-25)
Supplement: Additional file 5 — Table S1. Abbreviations for gene names. All the abbreviations for gene names used in this manuscript are listed. [file 1471-2350-13-25-S5.DOC]

**Supplemental Table S1**

Abbreviations for gene names.

| Gene symbol | Gene name |
| --- | --- |
| *A2M*  *ALOX5*  *B2M*  *CCL2/ 5/ 11/ 26*  *CCR4/ 5*  *CD14/ 28*  *CRP*  *CSF2*  *CTLA4*  *CX3CL1*  *EDN1*  *FASLG*  *HLA-C*  *ICAM1*  *IFNG*  *IGF1*  *IGFBP3*  *IL-1A/ 1B/ 2/ 4/ 6/ 8/ 11/ 12A/ 12B/ 15/ 17A/ 18/ 21/ 23A*  *IL-1R1/ 1RN/ 2RA/ 4R/ 6R/ 10RA/ 12RB2/ 18RAP/ 23R*  *IRAK3*  *IRF5*  *JAK2*  *KLRC1*  *LEP*  *MMP3*  *NFKB1*  *NFKBIA*  *NR3C1*  *PLAT*  *PPARA/ G*  *PTGS1/ 2*  *PTPN11*  *SELE*  *SERPINE1*  *SOCS1*  *SOD1/ 2*  *STAT2/ 3/ 4/ 6*  *TGFB1*  *TGFBR1*  *TIMP1*  *TLR1/ 2/ 4/ 6*  *TNFAIP3*  *TNFRSF1A/ 1B*  *TP53*  *TRADD*  *TYK2* | alpha-2-macroglobulin  arachidonate 5-lipoxygenas  beta-2 microglobulin  chemokine (C-C motif) ligand 2/ 5/ 11/ 26  chemokine (C-C motif) receptor 4/ 5  Cluster of Differentiation 14/ 28  C-reactive protein  colony stimulating factor 2  cytotoxic T-lymphocyte-associated protein 4  chemokine (C-X3-C motif) ligand 1  endothelin 1  Fas ligand  major histocompatibility complex class I C  intercellular adhesion molecule 1  interferon, gamma  insulin-like growth factor 1  insulin-like growth factor binding protein 3  interleukin-1 alpha/ 1 beta/ 2/ 4/ 6/ 8/ 11/ 12alpha/ 12beta/ 15/ 17A/ 18/ 21/ 23 alpha  interleukin-1 receptor 1/ 1 receptor antagonist/ 2 receptor alpha/ 4 receptor/ 6 receptor/ 10 receptor alpha/ 12 receptor beta 2/ 18 receptor accessory protein receptor 1/ 23 receptor  interleukin-1 receptor-associated kinase 3  interferon regulatory factor 5  Janus kinase 2  killer cell lectin-like receptor subfamily C member 1  leptin  matrix metallopeptidase 3  nuclear factor of kappa light polypeptide gene enhancer in B-cells 1  nuclear factor of kappa light polypeptide gene enhancer in B-cells inhibitor  nuclear receptor subfamily 3, group C, member 1  plasminogen activator tissue  peroxisome proliferator-activated receptor alpha/ gamma  prostaglandin-endoperoxide synthase 1/ 2  protein tyrosine phosphatase, non-receptor type 11  selectin E  serpin peptidase inhibitor, clade A member 1  suppressor of cytokine signaling 1  superoxide dismutase 1/ 2  signal transducer and activator of transcription 2/ 3/ 4/ 6  transforming growth factor, beta 1  transforming growth factor, beta receptor 1  TIMP metallopeptidase inhibitor 1  toll-like receptor 1/ 2/ 4/ 6  tumor necrosis factor, alpha-induced protein 3  tumor necrosis factor receptor superfamily member 1A/ 1B  tumor protein p53  TNFRSF1A-associated via death domain  tyrosine kinase 2 |
