# Supplementary material for: Systems analysis of inflammatory bowel disease based on comprehensive gene information
Source: BMC Med Genet. 2012 Apr 5;13:25. doi: 10.1186/1471-2350-13-25 (PMC3368714; doi:10.1186/1471-2350-13-25)
Supplement: Additional file 4 — Table S3. Bond order of susceptibility gene for IBD detected by GWAS. GWAS susceptibility genes [8,9] are listed with their bond order in HDNs of IBDmild and IBDsevere. [file 1471-2350-13-25-S4.DOC]

**Supplemental Table S3**

**Bond order of susceptibility gene for IBD detected by GWAS.**

GWAS susceptibility genes reported in are listed with their bond order in HDNs of *IBDmild* and *IBDsevere*.

| *IBDmild* | | *IBDsevere* | |
| --- | --- | --- | --- |
| gene | bond order | gene | bond order |
| *JAK2* | 17 | *IL12B* | 17 |
| *STAT3* | 14 | *IFNG* | 11 |
| *IL12B* | 13 | *IL23R* | 10 |
| *IFNG* | 10 | *IL2* | 9 |
| *IL23R* | 10 | *FASLG* | 4 |
| *IL2* | 10 | *IL21* | 4 |
| *CCL2* | 5 | *CCL2* | 3 |
| *IL2RA* | 5 | *IRF5* | 2 |
| *IL21* | 3 | *ICAM1* | 1 |
| *IL18RAP* | 2 |  |  |
| *ICAM1* | 1 |  |  |
| *IRF5* | 1 |  |  |

1. Franke A, McGovern DP, Barrett JC, Wang K, Radford-Smith GL, Ahmad T, Lees CW, Balschun T, Lee J, Roberts R *et al*: **Genome-wide meta-analysis increases to 71 the number of confirmed Crohn's disease susceptibility loci**. *Nat Genet* 2010, **42**(12):1118-1125.

2. Anderson CA, Boucher G, Lees CW, Franke A, D'Amato M, Taylor KD, Lee JC, Goyette P, Imielinski M, Latiano A *et al*: **Meta-analysis identifies 29 additional ulcerative colitis risk loci, increasing the number of confirmed associations to 47**. *Nat Genet* 2011, **43**(3):246-252.
